# RISC-y Business: Limitations of Short Hairpin RNA-Mediated Gene Silencing in the Brain and a Discussion of CRISPR/Cas-Based Alternatives

**DOI:** 10.3389/fnmol.2022.914430

**Published:** 2022-07-26

**Authors:** Kanishk Goel, Jonathan E. Ploski

**Affiliations:** ^1^School of Medicine, Texas Tech University Health Sciences Center, Lubbock, TX, United States; ^2^Department of Neural and Behavioral Sciences, Penn State College of Medicine, Hershey, PA, United States

**Keywords:** CRISPR, Cas9, Cas13, siRNA, shRNA, miRNA, CRISPRi, CasRx

## Abstract

Manipulating gene expression within and outside the nervous system is useful for interrogating gene function and developing therapeutic interventions for a variety of diseases. Several approaches exist which enable gene manipulation in preclinical models, and some of these have been approved to treat human diseases. For the last couple of decades, RNA interference (RNAi) has been a leading technique to knockdown (i.e., suppress) specific RNA expression. This has been partly due to the technology’s simplicity, which has promoted its adoption throughout biomedical science. However, accumulating evidence indicates that this technology can possess significant shortcomings. This review highlights the overwhelming evidence that RNAi can be prone to off-target effects and is capable of inducing cytotoxicity in some cases. With this in mind, we consider alternative CRISPR/Cas-based approaches, which may be safer and more reliable for gene knockdown. We also discuss the pros and cons of each approach.

## Introduction

DNA serves as the blueprint for all known life forms. Thus, the ability to manipulate gene function was long-sought-after because it could enhance our understanding of how individual genes contribute to biological systems. In 1989, Mario R. Capecchi, Martin Evans, and Oliver Smithies were the first to demonstrate that destroying or “knocking out” an individual gene in a mouse was possible. Due to the utility and importance of this technological advancement, they were awarded the Nobel Prize in Physiology or Medicine in 2007. Since then, the number of genetic tools or approaches enabling the manipulation of gene function has increased dramatically. One of these approaches, RNA interference or RNAi, knocks down a target gene’s expression by destroying its mRNA. This post-transcriptional interference strategy mimics the microRNA (miRNA) pathway that naturally exists in eukaryotic cells to regulate gene expression (Agrawal et al., 2003).

This article outlines the various drawbacks associated with transcriptome engineering using short hairpin RNA (shRNA) mediated RNAi techniques and highlights some new alternative technologies. We discuss multiple studies of the mammalian brain in which researchers have observed that the delivery of shRNA can cause unintended negative consequences. Ultimately, our discussion focuses on reducing the off-target effects of shRNA and exploring alternative methods for gene expression silencing.

## What Are MicroRNAs, Small Interfering RNAs, and Short Hairpin RNAs**?**

In 1993 the first small silencing RNA was discovered in the nematode *Caenorhabditis elegans.* This small RNA named lin-4 RNA could base pair with the *C. elegans* lin-14 mRNA and control the production of the LIN-14 protein (Lee et al., 1993; Wightman et al., 1993). This discovery was the beginning of a new era in biomedical science, where significant advancements enabled the genetic knockdown of virtually any mRNA. Lin-4 was the founding member of a relatively large class of genes that code for hairpin RNAs, now referred to as miRNAs. MicroRNAs consist of partially complementary double-stranded RNA, which are approximately 22 base pairs (bp) long and are responsible for halting genetic information flow in eukaryotes prior to translation (Cai et al., 2009). After a miRNA gene is transcribed by RNA polymerase II, it undergoes cleavage within the nucleus followed by export to the cytoplasm, where the Dicer protein removes its stem-loop to form the mature miRNA duplex (Lam et al., 2015). Then one of the miRNA strands binds to the RNA-induced silencing complex (RISC) and scans mRNAs for matching sequences prior to their processing. Once the miRNA/RISC binds to its target transcript’s 3′ untranslated region (UTR), it initiates mRNA degradation or inhibition of translation. There are over 2,500 miRNA genes within the human genome that are each predicted to regulate the expression of hundreds of genes, making miRNAs critical regulators of gene expression (Bartel, 2004; Griffiths-Jones et al., 2006).

**FIGURE 1:**
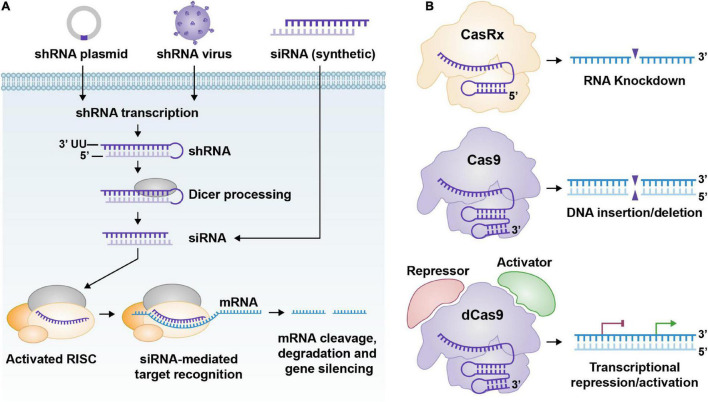
**(A)** General pathway for shRNA and siRNA; from delivery to processing. ShRNA expression cassettes are usually delivered to cells via DNA plasmids or recombinant viruses. Once the shRNA expression cassettes reach the cell nucleus, they can undergo gene transcription. The shRNA, once transcribed, is processed via Dicer and produces a functional siRNA. Synthetic siRNAs can be delivered to cells too. The siRNAs bind to the RISC complex and their target RNA and cleave their target RNA, which renders the RNA inactive. **(B)** Three graphical representations of common CRISPR technology. From top to bottom. CasRx (Cas13d)/gRNA complex can target specific RNAs for cleavage/destruction. Cas9/gRNA complex can bind to DNA and cleave it. This creates an insertion or deletion after the cell’s DNA repair machinery attempts to fix the DNA double-strand break. dCas9 fused to a transcriptional repression or activation domain (CRISPRi/a) complexed with a gRNA can reduce or enhance transcription of the target gene, respectively.

Not long after the first miRNA was discovered, it was found that double-stranded RNA could silence specific mRNA when introduced into cells (Fire et al., 1998; Hamilton and Baulcombe, 1999; Zamore et al., 2000). Double-stranded RNA of either exogenous or endogenous origin could be processed by Dicer in the cytoplasm to create small interfering RNA (siRNA) molecules, also known as short interfering RNA or silencing RNA (Figure 1). Silencing RNAs are non-coding RNAs spanning approximately 22 nucleotides that resemble miRNA and also serve to down-regulate gene expression post-transcriptionally. Silencing RNAs are derived from larger pieces of double-stranded RNA that result from viral infection, transposons, repetitive DNA elements, and DNA tandem repeats that are transcribed (Carthew and Sontheimer, 2009). Once siRNAs are processed by Dicer they are fed into the RISC complex similarly to miRNA. Unlike miRNA, however, siRNA’s guide strand is thought to require total complementarity to the target mRNA strand for successful binding and degradation (de Fougerolles et al., 2007). Scientists have adopted siRNAs to serve as one of the primary tools in RNAi. Synthetic siRNAs are commercially available for gene-specific targeting and have been widely adopted for gene knockdown. These synthetic siRNAs are usually introduced into cells via transfection in their mature, double-stranded form with overhangs. A potential limitation to this method of RNAi is that its effects are transient unless the siRNAs are replenished. This is because the siRNAs degrade over time. One way to mitigate the short-lived nature of standard RNA composed siRNAs is to create siRNAs composed of Locked Nucleic Acids (LNA) to increase their stability and half-life (Elmen et al., 2005; Mook et al., 2007; Vinnikov et al., 2014).

A more permanent way to reduce gene expression with RNAi is by introducing short hairpin RNA (shRNA) expression genes into cells. These genes are commonly introduced to cells using plasmids or viral vectors. These genes usually contain an RNA polymerase III promoter, such as the naturally occurring U6 promoter. This promoter drives the expression of the short RNA molecule consisting of two complementary regions of approximately 20 bp separated by a short stem-loop (Moore et al., 2010). This RNA spontaneously folds into a hairpin structure and is exported to the cytosol via exportin-5. Once in the cytoplasm, Dicer processes the shRNA to create a siRNA duplex. The antisense strand of the siRNA duplex ultimately binds to RISC and guides it to its target mRNA strand, slicing and feeding it into RNA decay pathways. The mature shRNA closely resembles a miRNA, with a characteristic hairpin structure containing four or more nucleotides, a 3′ overhang of two nucleotides, and a stem region spanning anywhere between 15 and 30 bp in length (Cullen, 2005; Kim et al., 2005; Ge et al., 2010). Advantages conferred by shRNA-mediated RNAi include its continuous expression and improved potency when compared to siRNA molecules targeting identical transcripts (McAnuff et al., 2007).

## Neurotoxicity and Off-Target Effects Due to the Expression of Short Hairpin RNAs

The viability of a gene-targeting technology greatly depends on its ability to accurately down-regulate its gene target without directly targeting unintended genes. However, shRNAs exert off-target effects in some cases (McBride et al., 2008; Hasegawa et al., 2017; Konermann et al., 2018; Czarnek et al., 2021). This is not entirely surprising considering that endogenous miRNAs found in mammals, which share similar processing and functionality to shRNAs, do not require complete complementarity to their targets and usually possess the ability to target multiple transcripts for degradation (Bartel, 2004). Thus, molecules that emulate their function likely retain this imprecision and versatility (Bartoszewski and Sikorski, 2019). In addition, shRNA expression in cells can potentially compete with endogenous miRNAs during all aspects of their processing, including their export from the nucleus, processing by Dicer, Argonaute, and RISC. These multiple steps create potential bottlenecks that could become saturated by shRNA expression, which leads to dysregulated gene expression since miRNAs are not able to function as intended (Grimm et al., 2006; Khan et al., 2009; Grimm, 2011; Baek et al., 2014; Dai et al., 2014).

Studies expressing shRNA in various rodent brain regions have observed off-target gene knockdown and shRNA-mediated cytotoxicity. In some cases, these unintended changes in gene expression have led to disturbances in neuron populations by influencing neuronal growth and maintenance. One study found that when shRNA was expressed in rat hippocampal pyramidal neurons, synapses and dendritic spines retracted (Alvarez et al., 2006). In this study, Short hairpin Luc (shLuc) was administered as a control shRNA designed to target the luciferase mRNA, which is not found in the mammalian genome. However, instead of acting as a non-targeting control shRNA, shLuc expression caused the gradual disruption of neuronal morphology. The expression of an alternative shRNA was insufficient to cause these dendritic and synaptic abnormalities suggesting saturation of endogenous miRNA pathways was not likely the cause. Instead, it was hypothesized that the seed region sequence of the control shRNA was partially complementary to transcripts critical to neurite maintenance. In another study, a variety of shRNAs induced cellular degeneration when delivered to the hippocampus (Gunther et al., 2017). The most substantial degeneration pattern in these experiments was in mice that received shLuc, raising concerns regarding the design of proper controls for RNAi experiments.

Transduction of shRNA control vectors *in vivo* has also been noted to elicit behavioral learning deficits in rodents. In a study examining the role of plasticity-associated genes in auditory Pavlovian fear conditioning, the infusion of control shRNA vectors into the basolateral amygdala unexpectedly thwarted fear conditioning in rats (de Solis et al., 2015). ShLuc perturbed fear learning and induced neurotoxicity, evidenced by microglial activation markers in the amygdala. This neurotoxicity depended on the adeno-associated virus (AAV) dose to deliver the shRNA gene. However, AAVs that did not harbor an shRNA gene did not induce any pathology or deficits in fear learning. Later, it was determined that shLuc was causing these aberrations in neural functioning, at least in part, by impairing voltage-gated ion channel function (Hasegawa et al., 2017).

Numerous other non-targeting shRNAs have been found to cause neuronal and synaptic degeneration in host cells. In a study aiming to silence mutant TorsinA mRNAs, which cause a neuromuscular condition known as primary dystonia, various intra-striatal shRNA injections (including non-targeting shRNA) proved lethal in all adult mice (Martin et al., 2011; Demircioglu et al., 2016). The mice which did survive, received AAVs lacking shRNAs. The shRNAs used in this experiment did not evoke neurotoxicity during previous *in vitro* studies. This discrepancy highlights difficulties in retaining shRNA safety and functionality across preclinical models. In another study of neurological disease, investigators determined that the delivery of shRNAs to the mouse striatum induced degeneration and death, and that reducing the dose of AAV-shRNA was insufficient to eliminate toxicity (McBride et al., 2008). Striatal toxicity likely arose from off-target effects of accumulated antisense shRNA products in the cytoplasm. Bypassing this accumulation and inducing efficient knockdown of target mRNA transcripts while avoiding cellular toxicity remains one of the most significant challenges in developing optimal shRNA expression cassettes.

Saturation of the miRNA pathway in the brain can alter the subject’s phenotype, leading investigators to improperly conclude that this change is due to shRNA on-target effects (van Gestel et al., 2014). Notably, discrepancies have occurred when a gene was knocked down vs. knocked out. For example, investigators observed neocortical neuronal migration defects when shRNAs were used to knockdown the doublecortin (Dcx) gene, but not when siRNAs or miRNAs were used to target the Dcx mRNA or when Dcx was knocked out in conventional knockout mice (Baek et al., 2014). The researchers concluded that shRNA overexpression led to alterations in the endogenous miRNA pathways. These findings indicate that loss-of-function experiments using shRNA should likely be revisited, given shRNA expression could disrupt endogenous miRNA pathways and target other transcripts. Table 1 summarizes notable discoveries of the referenced shRNA experiments (Table 1).

**TABLE 1:** Studies of shRNA delivery in various models and their respective observations.

Study	Key findings
Non-targeting shRNA expression in rat hippocampal pyramidal neurons (Alvarez et al., 2006)	•Reduced amount and strength of functional inhibitory and excitatory synapses. •Passive cell membrane properties (such as capacitance and resistance) impacted due to loss of neuronal membrane surface area.

shRNA expression in mouse primary hippocampal neurons (Gunther et al., 2017)	•Progressive hippocampal degeneration and tissue atrophy *in vivo* (strongest in mice injected with control shRNA, shLuc). •Increase in specific cellular stress markers found prior to shRNA induced neuronal degeneration. •No adverse effects when shRNA administered *in vitro.*

shRNA expression in rat basolateral amygdala (BLA) to assess effects on Pavlovian fear conditioning (de Solis et al., 2015)	•Auditory fear conditioning deficit demonstrated dose-dependence from shLuc shRNA levels. •Neurotoxicity detected in BLA due to shRNA overexpression.

Striatal injection of shRNAs to treat mouse model of primary dystonia (Martin et al., 2011)	•Differing levels of shRNA neurotoxicity between mouse strains (C57BL/6 and 129/SvEv). •Behavioral abnormalities, neuron loss, and lethality caused by shRNAs. •Delivery of same shRNAs to cultured cells did not result in cellular stress or death.

Striatal delivery of shRNAs to treat mouse model of Huntington’s disease (McBride et al., 2008)	•High levels of unprocessed shRNAs found *in vitro*, yet zero to minimal levels of unprocessed shRNAs seen *in vivo.* •Neurotoxicity in striatal tissues possibly due to off-target effects and cytosolic accumulation of antisense RNA. •Artificial miRNAs targeting same transcripts as shRNAs produced comparable degrees of transcript knockdown with superior safety.

Electroporation of various shRNAs causes defects in mice neuron migration (Baek et al., 2014)	•*In utero* cortical neuronal migration is compromised by various shRNA sequences; likely caused by saturation of endogenous miRNA pathway. •Normal neocortical migration noted in doublecortin gene knockout model, yet migration defects present in knockdown.

## Elements Contributing to Short Hairpin RNA’s Neurotoxicity and Poor Efficiency

Saturating the endogenous miRNA processing pathway is a potential cause of cytotoxicity following shRNA delivery. One way to mitigate this cytotoxicity is to select a suitable promoter for the gene construct containing shRNA. Opting for an RNA polymerase II promoter in shRNA transgenes has been seen to cause significantly less cytotoxicity than RNA polymerase III promoters, U6 (Giering et al., 2008). The U6 promoter is a potent inducer of transcription of its downstream transgene, leading to cellular stress and death if transcripts are not processed efficiently (Bish et al., 2011). This reduction in transcriptional potency of the shRNA expression cassette may sacrifice target mRNA degradation efficiency.

Scientists have found that designing shRNAs to be specific for their intended target is difficult. A study using human cells reported that an shRNA can target transcripts for degradation with as few as 11 nucleotides in common (Jackson et al., 2003). Additional studies deemed it impossible to eliminate off-target effects of shRNA-mediated transcript degradation because base pairing to a transcript requires only six nucleotides in the guide strand’s seed region (Birmingham et al., 2006; Ui-Tei, 2013; Bofill-De Ros and Gu, 2016). Thus, complications arise in designing an shRNA construct that binds solely to its targeted transcript. Another study found weaker base-pairing promoted specific binding between the guide strand and the target mRNA. While they failed to eradicate shRNA induced off-target effects, these experiments indicated that RNAi binding properties are more complex than previously considered (Gu et al., 2014).

## Alternatives to Short Hairpin RNA: Clustered Regularly Interspersed Short Palindromic Repeats/Cas Systems

Clustered regularly interspersed short palindromic repeats (CRISPR) associated protein (CRISPR-Cas) systems, adapted from the prokaryotic immune response, offer another way to control the expression of specific genes via genome and transcriptome manipulation. CRISPR/Cas9 genome editing can be reconstituted in eukaryotic cells simply by the presence of *S. pyogenes* Cas9 (SpCas9) protein and a guide RNA (gRNA)—just two genes are required. The first 20 nucleotides of the gRNA are custom designed to be complementary to the intended target site in the genome and consequently guide the Cas9 protein to this site, allowing Cas9 to create double strand breaks (DSB) in the genome. The only sequence requirements for Cas9, is that there needs to be a short sequence adjacent to the Cas9 nuclease cut site that the Cas9 molecule requires for appropriate binding. This sequence is referred to as the Protospacer Adjacent Motif (PAM sequence) and is composed of the nucleotides, NGG for Cas9 (Kleinstiver et al., 2015). Once the Cas9/gRNA complex docks to its target site and creates a DSB, the error prone non-homologous end joining (NHEJ) DNA repair mechanism is initiated. Due to the error prone nature of this repair pathway, insertions and deletions (Indels) are created at the DSB break/repair site. If the DSB occurs within the protein coding region of a gene, a loss of protein function can occur due to the deletion of relevant codons, or a shift in the reading frame can occur, often creating a truncated protein—collectively leading to a gene knockout (Jinek et al., 2012; Cong et al., 2013; Mali et al., 2013b). Alternatively, if a donor DNA template is provided, Homology Directed Repair (HDR) can occur instead of NHEJ. This phenomenon can be harnessed to create specific modifications of the genome at very precise loci (Cong et al., 2013; Mali et al., 2013b; Wang et al., 2013).

Delivering an expression cassette coding for a guide RNA and its associated Cas endonuclease protein, have allowed neuroscientists to disrupt the genome in neurons and knock out genes to screen for their function (Incontro et al., 2014). RNA-guided double-stranded DNA cleavage with CRISPR has also offered a route to introduce (or knock-in) functional genes using templates that compensate for deleterious mutations (Yang et al., 2013). Both knock-in and knockout mechanisms may give rise to therapeutic approaches for different diseases based on their varying pathogeneses.

Since the discovery of CRISPR-Cas, the technology has been adapted for many genomic applications such as gene knock-in, transcriptional activation/repression, RNA degradation, epigenetic modification, and DNA/RNA base editing. A common theme with CRISPR is that Cas enzymes are proteins that are guided to specific DNA or RNA loci via a gRNA. Because of this ability, Cas enzymes have also been rendered catalytically inactive and modified to contain additional protein domains that allow locus-specific manipulations. For example, the catalytically inactive nuclease deficient SpCas9D10A/H841A mutant (dSpCas9), when coupled with a gRNA targeting a promoter/5′UTR region can lead to transcriptional repression of that gene essentially creating a reversible gene knockout. However, it was found that the degree of transcriptional repression could be significantly enhanced if dSpCas9 was fused to the transcriptional repressor domain [Krueppel-associated box, (KRAB)]. Similarly, it was found that gene specific transcriptional activation could occur if dSpCas9 was fused to the transcriptional activator domain, VP64. These two strategies to bi-directionally manipulate gene expression are referred to as CRISPRi and CRISPRa, respectively (Gilbert et al., 2013; Larson et al., 2013; Qi et al., 2013; Tanenbaum et al., 2014). Since the inception of CRISPRi/a, there have been modifications to these systems to improve their ability to suppress and activate gene expression (For review, see Sandoval et al., 2020).

CRISPR-Cas13 subtypes have also enabled the exclusive targeting of cellular RNA, offering the desired outcome similar to that of RNAi while avoiding the saturation of the miRNA pathway and its associated neurotoxicity (Hale et al., 2009; Abudayyeh et al., 2017; Smith et al., 2017). The guide RNA in this technique contains a programmable sequence spanning approximately 20–30 nucleotides, that when bound with Cas13, can guide Cas13 to its target RNA where the Cas13 ribonuclease cuts the RNA leading to its destruction. Since the programmable sequence is approximately three times larger than the seed sequence of shRNA, CRISPR-Cas mediated post-transcriptional silencing grants researchers greater target strand specificity. Another benefit is that Cas13 doesn’t require a PAM sequence, allowing it in theory to target virtually any region of an RNA. However, not all CRISPR Cas13 systems are created equal. Variability in performance has been seen within CRISPR-Cas13 subtypes, with CasRx (also known as Cas13d) significantly outperforming their Cas13a and Cas13b counterparts in transcript knockdown assays (Konermann et al., 2018).

## Fidelity and Efficiency of Clustered Regularly Interspersed Short Palindromic Repeats/Cas Systems

Similar to RNAi, CRISPR/Cas systems need to be actively vetted for their specificity and safety profile in cells and animals. SpCas9 mediated genome editing is reasonably accurate, but a number of studies have demonstrated that Cas9 can bind and induce DSBs at sites that are not entirely complementary to the gRNA sequence (Fu et al., 2013; Hsu et al., 2013; Pattanayak et al., 2013). Because of this, ways to improve the fidelity of genome editing have been actively pursued. Cas9 mediated off-target editing can be reduced by limiting the time Cas9 or the gRNA remains active in cells. This can be accomplished through the use of self-inactivating Cas9 vectors (Moore et al., 2014; Ma et al., 2022), or by the use of inducible expression systems that transiently express Cas9 or the gRNA (Davis et al., 2015; Zetsche et al., 2015; de Solis et al., 2016). Other approaches are to use the Cas9 nickase to create two single stranded cuts close together on opposing DNA strands (Mali et al., 2013a; Ran et al., 2013). Improving gRNA design by truncating the guide sequence at the 5′ end has also been shown to enhance fidelity (Fu et al., 2014). Alternatively, efforts have also been made to mutate Cas9 and select for variants that exhibit higher fidelity while maintaining efficient on-target nuclease activity. High-fidelity variants of SpCas9 include eSpCas9 (1.1) (Slaymaker et al., 2016), SpCas9-HF1 (Kleinstiver et al., 2016), HypaCas9 (Chen et al., 2017), evoCas9 (Casini et al., 2018), and Sniper-Cas9 (Lee et al., 2018). A recent study ranked their overall activity in the following order: SpCas9 ≥ Sniper-Cas9 > eSpCas9 (1.1) > SpCas9-HF1 > HypaCas9 ≈ xCas9 > > evoCas9. But for the most part, there is an inverse relationship between the ability of the Cas protein to induce DSBs vs. its accuracy. Their overall specificities are ranked as evoCas9 ≫ HypaCas9 ≥ SpCas9-HF1 ≈ eSpCas9 (1.1) > xCas9 > Sniper-Cas9 > SpCas9 (Kim et al., 2020).

CRISPRi/a has been found to be remarkably accurate, exhibiting minimal off-target effects, while maintaining the ability to robustly manipulate gene expression (Gilbert et al., 2013, 2014; Qi et al., 2013). However, off-targeting does still occur (Daley et al., 2018; Stojic et al., 2018). Whole genome library screens have shown that gRNAs targeting bidirectional promoters can lead to false positives (Rosenbluh et al., 2017). Improvements in gRNA design strategies can allow the selection of gRNAs that exhibit minimal off-target effects, with maximal on-target effects. For example, the development of screening algorithms such as GuideScan, CHOPCHOP, and DeepHF have significantly improved the ability to select appropriate and effective gRNAs (Perez et al., 2017; Wang et al., 2019a; Labun et al., 2021).

Some comparisons of shRNA and CRISPR-Cas9 systems outlined thus far are available for review. One study presents a side-by-side comparison of CRISPR-Cas9 and shRNA screens for essential genes in K562 cells. It was found that though both approaches were precise, the Cas9 system identified many more critical genes, and the two screening technologies identified different categories of genes (Housden and Perrimon, 2016; Morgens et al., 2016). Another study compared CRISPR-Cas9, CRISPRi, and shRNA technologies in their ability to screen for essential genes. They found that CRISPR technology performed best, with low noise and minimal off-target effects (Evers et al., 2016). Other surveys using CRISPR systems for essential and non-essential human genes detection revealed that previous identification of these genes through RNAi reverse screens was largely deficient (Wang et al., 2015; Evers et al., 2016). While this comparison in sequence recognition and destruction provides a strong case for CRISPR’s superiority, it may not be wise to phase out RNAi just yet. This is because genetic screens using a combination of CRISPR and shRNA provide better results than either approach alone (Deans et al., 2016).

Some researchers believe that shRNA off-target effects are impossible to evade (Ui-Tei, 2013). Unfortunately, shRNA off-target effects are not the only ones that require improvement, as disappointing on-target effects also raise questions about shRNA’s interrogative and therapeutic potential. CRISPRi was shown to significantly outperform shRNAs in cultured hippocampal neurons when attempting to knockdown several genes necessary for neurotransmission. CRISPRi was able to knockdown the genes by approximately 90%, while shRNA mediated knockdown was much less efficient (Zheng et al., 2018).

CRISPR-CasRx significantly outperformed shRNA (96% vs. 65%) when comparing their ability to knockdown three genes (Konermann et al., 2018). This study also noted that 900 off-target effects were witnessed using the shRNA technique, whereas competing CasRx approaches resulted in zero off-target events. In many ways CasRx appears superior to RNAi. Numerous reports have also shown that CasRx can be successfully used in human cells (Konermann et al., 2018), mice (Zheng et al., 2022), and zebra fish (Hernandez-Huertas et al., 2022), with no apparent cytotoxicity. However, one study found that CasRx caused *in vivo* toxicity to Drosophila melanogaster flies (Buchman et al., 2020). Several other recent studies using mammalian systems, have now shown that when Cas13 (including CasRx) engages in gRNA dependent on-target RNA cutting, it also engages in gRNA independent collateral RNA targeting. It randomly cuts RNAs that are in close proximity to the Cas13 enzyme when it is cutting its intended target, with highly abundant RNAs at most risk (Wang et al., 2019b; Shi et al., 2021; Ai et al., 2022; Li et al., 2022a,b). When CasRx was expressed in mouse neurons, it led to the collateral cleavage of the 28s rRNA and induced death of the mice. This is extremely disappointing, considering that for an otherwise great tool, the collateral RNA targeting of CasRx disqualifies it as an appropriate technology to use for preclinical or clinical purposes. In better news however, a soon to be published study identified variants of CasRx that possess minimal collateral cutting activity (Tong et al., 2021). Hopefully this new variant will live up to expectations.

With the advent of CRISPR technology and its many improvements and modifications, RNAi approaches have been eclipsed (Unniyampurath et al., 2016). The limited advantages of shRNA methods continue to wane as CRISPR techniques advance. This trend will likely continue as the paradigm for legitimate gene-editing tools becomes more rigid in its expectations of efficiency and off-target effects. Nowhere is this more applicable than in the development of gene therapies, where off-target results may prove fatal for patients. The advantages and limitations of both RNAi and CRISPR-Cas tools for gene manipulation are presented in Table 2.

**TABLE 2:** Pros and cons of various RNAi and CRISPR-Cas gene manipulation techniques.

Gene manipulation	Pros	Cons
shRNA	•Biochemical machinery (DICER and RISC) for processing already present in cells. •Established shRNA libraries are already available for specific gene silencing across entire genomes. •Rapid interference and high throughput. •Capable of assessing essential gene function.	•Significant amount of off-target effects. •Associated with immune response and cytotoxicity. •Unsatisfactory knockdown efficiency of target mRNA strand. •May compete with endogenous miRNA pathway.

siRNA	•Chemical modification of siRNA is simpler than altering components of plasmid and vector design in other techniques. •Biochemical machinery (RISC) for action already present in host cells. •Requires minimal intracellular processing for maturation and action. •Capable of assessing essential gene function.	•Significant amount of off-target effects. •Associated with immune response and cytotoxicity. •Less efficient than shRNA in target knockdown. •May compete with endogenous miRNAs for RISC. •Rapidly cleared by host organism. •100-fold decrease in cytosolic concentration 48 h after administration. •Requires recurring administrations for long-term transcript silencing, unless siRNAs are created with modified nucleotides.

CRISPR-CasRx	•More precise base pairing to target mRNA strand due to larger mRNA complementarity region. •Fewer off-target effects than RNAi techniques. •Greater knockdown efficiency than RNAi techniques. •Robust design tools available for sgRNA creation. •Does not compete with miRNA for cellular machinery. •Capable of assessing essential gene function.	•*In vivo* toxicity observed in *Drosophila melanogaster*. •gRNA independent collateral cutting of cellular RNAs significant issue. •Requires more *in vivo* studies to reinforce safety and efficacy in humans.

CRISPRi/CRISPRa	•Highly specific and reversible means of transcriptional repression or activation. •Very few off-target effects. •Allows fine tuning of target gene expression levels. •Does not need to alter target sequence DNA to down or upregulate its gene products. •Does not compete with miRNA cellular machinery. •Targets long non-coding RNAs without multiple sgRNAs. •Capable of assessing essential gene function.	•Cannot reduce target gene expression to zero. •May influence transcription of adjacent genes.

CRISPR-Cas9	•Design tools are available to create sgRNA sequences. •Improvements in off-target effects due to advancements in sgRNA design tools and availability of Cas9 high fidelity variants. •Rapidly expanding accessibility to pooled CRISPR guide RNA libraries. •Permanent gene silencing avoids potential confounding results from trace gene products. •Less expensive means of gene manipulation. •Allows knocking-in of exogenous genes using homologous recombination of DNA. •Capable of assessing essential gene function.	•Safe translation of CRISPR-Cas9 into *in vivo* human disease models requires more investigation. •Close to 1/3 of insertions/deletions fail to knockout target gene via frameshift mutation.

## Conclusion

Transcriptome engineering has accelerated in the past two decades and will continue to do so in the coming years, as the FDA approval of transcriptome regulating gene therapies has already begun. CRISPR-Cas systems require additional improvements in design but indicate more significant potential in biomedical applications when compared to RNA interference technologies. CRISPR technologies have been rising in popularity due to their proven versatility, efficiency, and limited off-target effects. On the other hand, factors contributing to the shRNA technology’s neurotoxicity and poor efficiency make it highly unlikely that the system will see significant improvements in the coming years. Advancements in shRNA design will have to balance the burdens of efficiency and safety, theoretically progressing toward low doses of highly specific shRNA, which effectively knockdown target transcripts. These advancements have many challenges, including proper control shRNA design and refined comprehension of off-target transcript binding mechanisms.

When investigating *in vitro* models of neurological disease, researchers will continue to witness greater efficiency and accuracy from CRISPR-Cas systems than shRNA and other RNAi approaches. The broader medical adoption of CRISPR systems may be accelerated as their *in vivo* safety improves alongside the stability of delivery vehicles. As the first clinical trials examining CRISPR technologies have launched in the past year, their pending results will greatly influence the future direction of clinical genome and transcriptome editing. Beyond the ongoing human trials, personalized gene therapies will undoubtedly continue to revolutionize medicine and neuroscience in the coming decades, offering solutions to countless conditions at the chromosomal level.

## Author Contributions

KG wrote the original draft of the manuscript. JP edited the manuscript with feedback from KG. Both authors contributed to the article and approved the submitted version.

## Conflict of Interest

The authors declare that the research was conducted in the absence of any commercial or financial relationships that could be construed as a potential conflict of interest.

## Publisher’s Note

All claims expressed in this article are solely those of the authors and do not necessarily represent those of their affiliated organizations, or those of the publisher, the editors and the reviewers. Any product that may be evaluated in this article, or claim that may be made by its manufacturer, is not guaranteed or endorsed by the publisher.
